# Challenges, perceptions, and future preferences for post-secondary online education given experiences in the COVID-19 outbreak

**DOI:** 10.1007/s43762-022-00058-7

**Published:** 2022-08-30

**Authors:** Hamidreza Asgari, Rajesh Gupta, Ibukun Titiloye, Xia Jin

**Affiliations:** 1 Department of Civil and Environmental Engineering, Florida International University, 10555 W. Flagler Street, EC3725, Miami, FL 33174 USA; 2 Department of Statistics, University of Lucknow, University Rd, Lucknow, Uttar Pradesh 226007 India; 3 Department of Civil and Environmental Engineering, Florida International University, 10555 W. Flagler Street, EC 3603, Miami, FL 33174 USA

**Keywords:** Online education, Perceptions and experiences, Structural equation model, Covid-19

## Abstract

To gain a better understanding of online education status during and after the pandemic outbreak, this paper analyzed the data from a recent survey conducted in the state of Florida in May 2020. In particular, we focused on college students’ perception of productivity changes, benefits, challenges, and their overall preference for the future of online education. Our initial exploratory analysis showed that in most cases, students were not fully satisfied with the quality of the online education, and the majority of them suffered a plummet in their productivities. Despite the challenges, around 61% believed that they would prefer more frequent participation in online programs in the future (compared to the normal conditions before the pandemic). A structural equation model was developed to identify and assess the factors that contribute to their productivity and future preferences. The results showed that lack of sufficient communication with other students/ instructor as well as lack of required technology infrastructure significantly reduced students’ productivity. On the other hand, productivity was positively affected by perceived benefits such as flexibility and better time management. In addition, productivity played a mediating role for a number of socio-economic, demographic, and attitudinal attributes: including gender, income, technology attitudes, and home environment conflicts. Accordingly, females, high income groups, and those with home environment conflicts experienced lower productivity, which indirectly discouraged their preference for future online education. As expected, a latent pro-online education attitude increased both the productivity and the future online-education preference. Last but not the least, Gen-Xers were more likely to adopt online-education in the post pandemic conditions compared to their peers.

## Introduction

The unprecedented level of threat that Covid-19 poses to the health and well-being of the entire global population has led to emergency government actions, one of which is the closure of educational facilities in more than one hundred countries (Nicola et al., 2020). In the U.S., institutions were given few days’ notice to prepare for the cancellation of all in-person classes and switch to a remote delivery format to maintain “social distancing” guidelines. This situation has forced all levels of the educational system to adopt online emergency remote teaching. As of May 2020, the percentage of staff in the education industry working completely from home had risen from 4.6% in February to 58.9%, and yet, there was further potential for additional home-based work in the education industry (Bick et al., 2020).

There is a tendency to equate this emergency mode of teaching to online education, however, they are not the same. Noted differences between this emergency mode of teaching and online education have caused some researchers to label it as “emergency remote teaching” (Hodges et al., 2020). It was defined as “a temporary shift of instructional delivery to an alternate delivery mode due to crisis circumstances”. The temporariness of the delivery mechanism of emergency remote learning, and the time constraints required to make courses and instructional support system accessible online make time factor the primary component that differentiates it from regular online programs. In the case of online education, there is an uncoerced decision to plan with stakeholders, vet resources, train faculty, and develop instructional support system through collaboration between teachers and technology specialists. However, emergency remote teaching involves making forced decisions. Instructors must assess whether syllabus meets students’ and instructors’ needs in terms of technology, workload, access, accessibility, equity, and inclusion. The constraint of working from home must then be considered in modifying course design, tools, and delivery formats (Gacs et al., 2020). Moreover, training and support are necessary and must be done under time pressure. Where planning and preparing for a fully online university course may take up to 9 months, the time allowance in transitioning to emergency remote teaching may be limited to a few weeks only or even shorter time. Often, frustration of instructors, burden on resources, and the time constraints necessitate compromises in key areas that inevitably diminish the quality of teaching.

Some of the well-documented challenges of online education may apply to emergency remote teaching as well. For example, critics of online education have argued that online education has failed to improve affordability, does not yield a positive return on investment, has not lived to its promise to increase authentic accessibility, and has widened gaps in educational attainment across socio-economic groups than in traditional coursework (Lee, 2015, 2017; Protopsaltis & Baum, 2019; Tucker, 2007). It has been noted that substantial digital inequalities exist, as one out of every five of low- and moderate-income families connect to the Internet only through a mobile device (Rideout & Katz, 2016). Also, students of low socio-economic status are more likely to bear the cost of achieving access to technology, tend to have poorly functioning and frequently failing laptops, and have worse academic performance than students of high socio-economic status (Gonzales et al., 2020). Thus, students who may find emergency remote teaching more challenging are those from low- and moderate-income families.

Also, lack of substantive interactivity among students and between students and faculty, which seem to be the major shortcoming of online education (Lee, 2017; Protopsaltis & Baum, 2019) may also apply to emergency remote teaching. Diminished quality of teaching coupled with instructors’ unfamiliarity with technologies, and overburdened resources may worsen the challenge of lack of interactivity, and thus reduce student satisfaction. Other challenges may include students’ frustration due to a lack of self-regulated learning skills and various time management issues (Lee et al., 2019), inability to appeal to far-located students (Palvia et al., 2018), students’ need for higher metacognitive skills and additional multitasking skills for success, and higher dropout rates among online learners as compared to traditional face-to-face students (Lee, 2015).

Understanding students’ perceptions and experiences in emergency remote teaching environments would be necessary as there seems to be growing expectations that the disruption done to education may lead to a decade-long technology-led remaking (Krishnamurthy, 2020). Predictions of permanent disruptions are reasonable, especially when one considers that 98% of “Education, Training, and Library Occupations” in the U.S. can plausibly be performed from home (Dingel & Neiman, 2020). The experiences during COVID-19 provide a unique opportunity for universities and colleges to identify the strengths and challenges of their emergency remote teaching programs, and to better prepare for the future. It also may shed some lights on the perceptions and expectations for regular online programs. Given this motivation, this study aims to explore the perceptions and experiences of students on various aspects of emergency remote teaching in the wake of the Covid-19 pandemic. Authors believe that the analysis of real-life experiences gained during the pandemic distant-learning practice can unveil valuable insights to the researchers and planners, which can result in better preparation and more efficient and feasible strategies by taking into account students’ perceptions and challenges.

An online survey was conducted in May 2020 to collect information from college students. Using the survey data, this study evaluated various attitudinal aspects of students in response to emergency remote teaching. The main objective of this study in big picture is to assess students’ perceptions toward different aspects of online education, including their previous experience, productivity, education quality, and factors that affected their distant learning experience. In addition to a comprehensive exploratory data analysis equipped with statistical tests, a predictive analytical framework was also constructed, using structural equation model. In particular, our model explored the causal effects from a variety of personal traits and attributes on students’ productivity during the pandemic and on their future preferences for online programs.

The remaining of the paper lays out as the following: in the next section we provide a brief review of the existing literature in view of online education experiences and the role of different contributing factors in terms of adoption, productivity, attitudes and well-being of students. We then briefly elaborate our data collection approach as well the sample dataset statistics and explore the existing statistical relationships as well as their significance among a number of education-related parameters. Model theory and methodological details come next, followed by our model results. We further delve into some potential policy implications of our findings. Las but not the least, we conclude our paper by presenting the highlights, laying down the study limitations, as well as providing recommendations for future research.

## Literature review

This section presents findings from studies that have explored the perceptions and experiences of students, faculty members and academic leaders to online education. Studies that sought to identify factors that affect online students’ academic success have also been included. This section ends with the few studies done on perceptions of individuals to emergency remote teaching.

Several studies have documented students’ perceptions of online education in recent years. Different terms and metrics have been used by researchers to evaluate the success of online educational programs from students’ perspectives. In general, it is evident that communication (both with the instructor and other students), organizational support, accessibility and user-friendliness of content delivery, as well as familiarity with the technology played important roles in educational efficiency and students’ satisfaction.

Antoine (2011) showed that the flexibility of the program and mode of delivery were the primary factors that influenced students’ decision to enroll in online programs. Furthermore, the psychological needs of students were met through the enablement to achieve a degree that was otherwise seen as unattainable. The study also pointed to the critical role of good leadership (e.g., teachers and support services) especially when students were still somewhat unfamiliar with the requirements, technology, and functional elements of the online program setting. Ilgaz and Gülbahar (2015) assessed the quality of e-learning by conducting a before-after survey on students’ online learning experience. It was found that students’ choice of e-learning was related to participants’ accessibility and individual responsibilities, while students’ satisfaction was influenced by instructional content (the most influential factor), communication and usability, and teaching process. Ragusa and Crampton (2018) explored the role of “connection” and “identity” in online students’ academic success. It was found that self-identity positively correlated with sense-of-connection, though connection varied by course. While students described distance education as isolating, those who studied more reported that they felt more connected to their course, lecturers, and classmates. Moreover, the quality and timeliness of lecturer feedback may be more important to students than technological sophistication. Lee et al. (2019) conducted a study on 10 adult distant learners in an undergraduate program and suggested that online students would need more support and structure, require sharing useful knowhows with other students, and find multiple pedagogical choices and options as being confusing and stressful. In a similar study, Alqurashi (2019) inferred that student satisfaction rates would increase if online course materials were easily accessible, stimulate students’ interest, helps them understand the class content, and relate their personal experiences to the new knowledge gained. An open-ended survey by Muir et al. (2019) showed that individuals’ weekly engagement in online education was influenced by assessment tasks, workload across units, nature of the units (including delivery and relevance), presence of and relationship with lecturer, and other life commitments. Moreover, student-faculty interaction was valued more than peer interaction through discussion boards. Similarly, Stone (2017) emphasized on teacher-presence, regular and structured contact between the institution and the student, as well as proactive follow-ups with students.

Some studies have stepped further and explored the socio-economic and demographic heterogeneity across adult online students (Muljana & Luo, 2019). Speaking of gender, mixed results are observed. Yoo and Huang (2013) showed that female students had a stronger intrinsic motivation to take online courses than their male counterparts. Similarly, Cochran et al. (2014) showed that males were more likely to withdraw from online courses. In view of performance, females were reported to be weaker in online courses, though the inference is limited to STEM fields. No significance gender impact was observed according to Eliasquevici et al. (2017). Another variable discussed in the literature is age. According to Wuellner (2013), younger students were more likely to lack the required maturity and readiness for online learning. Furthermore, Wladis et al. (2015) showed that older students had better performance in online courses. Similar results were documented by Yoo and Huang (2013) as participants in their twenties, thirties, and forties reported a higher level of relevance in their short-term and long-term extrinsic motivation than the rest of the age groups. Results are more or less in line with previous research findings, having documented an overall better academic performance by older students, with full-time enrollment status, and with more educational experience (Colorado & Eberle, 2010; Guri-Rosenblit, 1999; Moore & Kearsley, 2005; Nesler, 1999).

Some other researchers have looked into “Emergency remote teaching”. The term was initially coined by Hodges et al. (2020) in the wake of Covid-19. Since this subject area is a very novel one, the literature addressing this area of study is limited. Before the Covid-19 disease was declared a pandemic by the W.H.O., Jaschik and Lederman (2020) conducted a quantitative survey research study of 746 university presidents to examine how they viewed pressing issues facing higher education in the U.S. Though less than half (45%) of respondents believed their college had the right tools and processes to adapt to needed change, more than half (54%) of them believed their college had the right mindset. More recently in March 2020, a survey conducted by Ganesh Kumar et al. (2020) was also administered to university and college presidents to understand their perceptions in the wake of Covid-19. It was observed that 89% and 88% of respondents were “very concerned” or “somewhat concerned” about the overall financial stability and decline in overall future student enrollment, respectively. Also, 53% of them felt their institution needed support in faculty training and development, 48% in instructional technology development, and 41% in student support services. In another study, Händel et al. (2020) explored the e-readiness of higher education students for emergency remote learning and how it affected the students’ socio-emotional experiences. The study was conducted among 1826 higher education students in a German university. Results showed that students who were ready for digital learning reported less tension, overload, worries, social and emotional loneliness, but higher joy and better work life balance.

## Data collection

In order to gain a deeper understanding of the pandemic effects on college students’ learning experience and their perceptions of and attitudes toward emergency remote teaching, an online survey was conducted in May 2020 in South Florida, right before the initiation of the reopening phases. The survey collected information on personal attributes, activities, experiences, preferences, and attitudes related to remote learning. To evaluate the impact of COVID-19, both past and current experiences with online learning were recorded. In addition, to assess the long-term impacts of the pandemic, respondents were also asked about their expected behavior after the COVID-19 is no longer a threat. This information would provide additional insights on how much of the observed patterns during the pandemic may influence individual’s behavior in the long term.

Qualtrics online platform was employed for survey implementation and recruitment. Data were collected between May 19th and May 29th, 2020. Responses from 363 college students in south Florida were collected, including 241 (66%) full-time and 121 (33%) part-time students. The student sample consisted of 58.6% male and 41.4% females, and around 31% were married. In view of ethnicity, 65.5% claimed to have Hispanic roots. 61.4% were recognized as white and 22% were Black or African Americans. Speaking of education, 18.6% were high school graduates, 60.7% were undergrads (including associate and professional degrees), while a total of 17.2% held postgraduate degrees (13.5% Masters’ and 3.7% Doctorate). Around 74% of the students were employed before the pandemic, either as full-time (43%) or part-time employed (31%), compared to a total of 68% currently employed (during COVID-19).

## Exploratory data analysis

This section presents explorative data analysis focusing on the patterns as well as perceptions and attitudes toward remote learning during the pandemic. Several cross tabulations as well as contingency chi-square tests, t-tests, and proportional z tests were conducted.

**Fig. 1:**
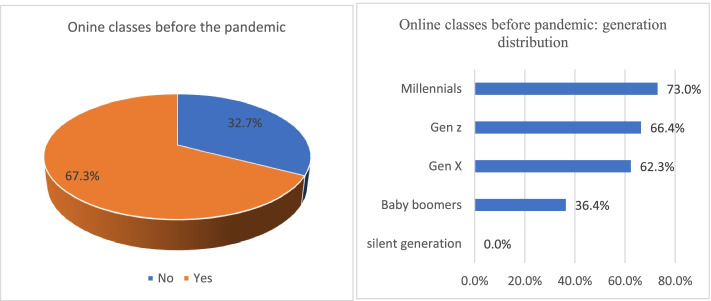
Online class participation before Covid-19

Around 67% of students said they were already taking online classes before COVID-19, as shown in Fig. 1. There was significant association between generation and prior experience of online classes. Accordingly, 73% of Millennials were taking online courses before COVID-19, followed by 66.4% of Generation Z and 62.2% of Generation X (*p*-value: 0.02). No statistically significant association was observed with other socio-demographic factors including gender, marital status, ethnicity, race, education and income (*p*-value not significant).

**Fig. 2:**
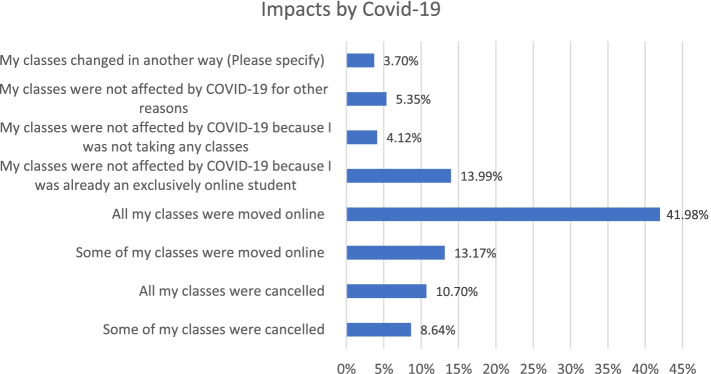
Schedule Changes by Covid-19

In view of changes imposed by the pandemic as shown in Fig. 2, most of the students (42%) said all classes were moved to online, about 14% were not affected because they were already exclusively online students. About 10% students had all their classes cancelled.

**Fig. 3:**
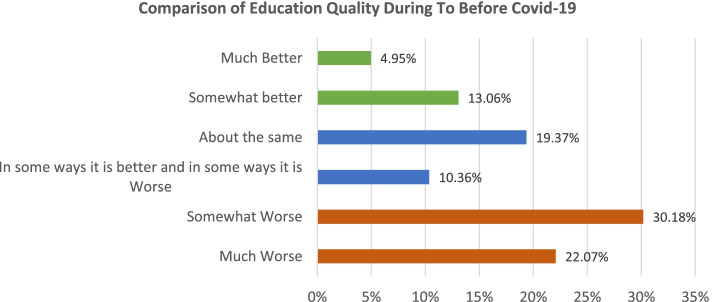
Comparison of education quality compared to before the pandemic

Respondents were also asked about the quality of remote education compared to regular conditions before the pandemic. As shown in Fig. 3, the results did not seem satisfactory as 52% of the respondents stated that the education quality was somewhat worse or much worse for online classes now compare to before COVID-19.

Cross tabulation results showed that the responses regarding education quality were highly heterogeneous across the education levels. Around 75% with ‘less than high school’ education said much worse, followed by 60% doctorate while 57% associate degree people says ‘much worse’ or ‘somewhat worse’ [*p*-value: 0.022]. Another contributing factor was marital status being marginally associated with online classes satisfaction (*p*-value: 0.049). Interestingly, divorced and widowed individuals showed the highest percentages stating their education quality was somewhat better than normal conditions (75% and 50%, respectively). No significant association was detected with other factors including ‘Gender’, ‘Hispanic’, ‘Race’, and ‘Income class’ (*p*-value not significant).

**Fig. 4:**
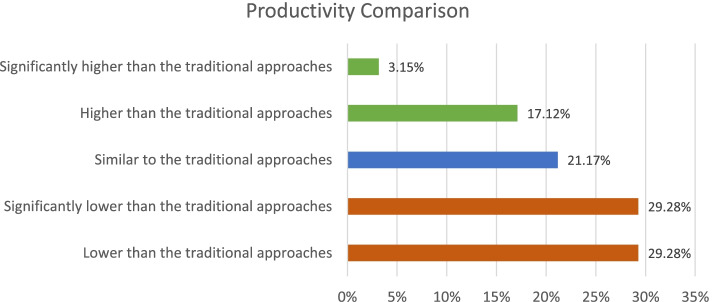
Productivity during the pandemic compared to regular approach

Quality perception is expected to have a direct relationship with productivity. Accordingly, 60% of the students said their productivity was lower or significantly lower than traditional approaches compare to before COVID-19 as shown in Fig. 4. No association was observed between productivity change and demographic factors.

**Fig. 5:**
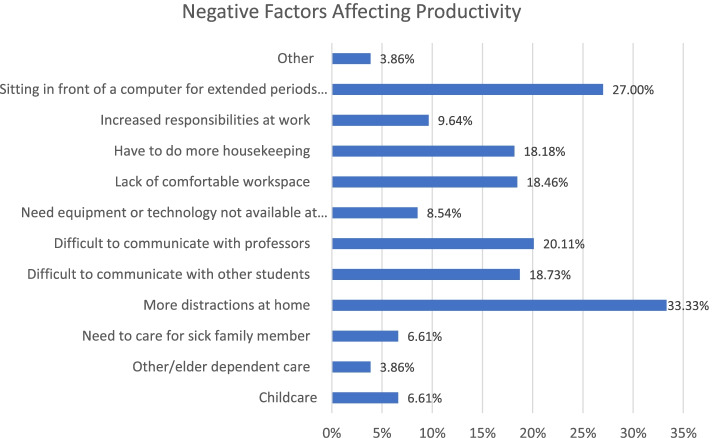
Factors negatively affecting productivity

When asked about productivity changes, individuals were also inquired about the factors that encouraged or discouraged their productivity in view of online education. ‘More distraction’, ‘Difficult to communicate with other students’, ‘Difficult to communicate with professors’, ‘Lack of comfortable workspace’ and ‘More housekeeping work’ were the main factors affecting productivity negatively, as shown in Fig. 5.

**Fig. 6:**
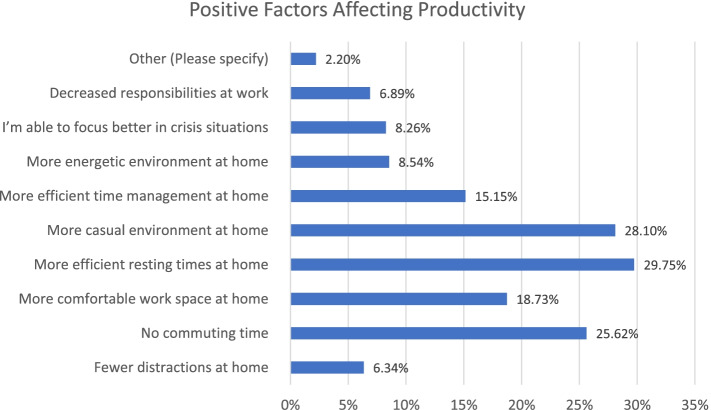
Factors positively affecting productivity

On the contrary, ‘More efficient resting time’, ‘More casual work environment at home’ and ‘No commuting time’ were the main factors for that affect productivity positively, as shown in Fig. 6.

**Fig. 7:**
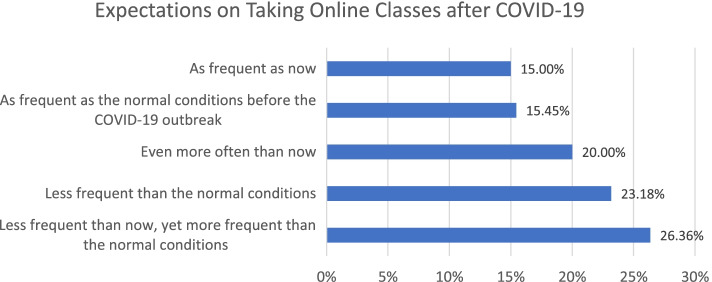
Preferences for taking online classes after COVID-19

In view of their preferences after COVID-19 is no more a threat, Fig. 7 shows that around 26% of the respondents said they would prefer taking less frequent online classes yet more frequent than before COVID-19, while 23% preferred taking less frequent online courses even than before COVID-19. 20% were interested in taking online classes even more often.

Statistical tests revealed that the future preferences were highly correlated with Generation (*p*-value: 0.001) and marital status (*p*-value: 0.03). Gen-Z (31%) and Millennials (27.6%) showed the highest percentages of ‘less frequent than now but more frequent compared to normal conditions before Covid-19’. In view of marital status, divorced individuals showed the highest percentage of ‘taking less frequent online classes even compared to normal conditions’ (75%), while married respondents reflected the highest percentage of maintaining similar frequency as they did before (21.8%) or even more frequently (30.9%).

## Methodology

**Fig. 8:**
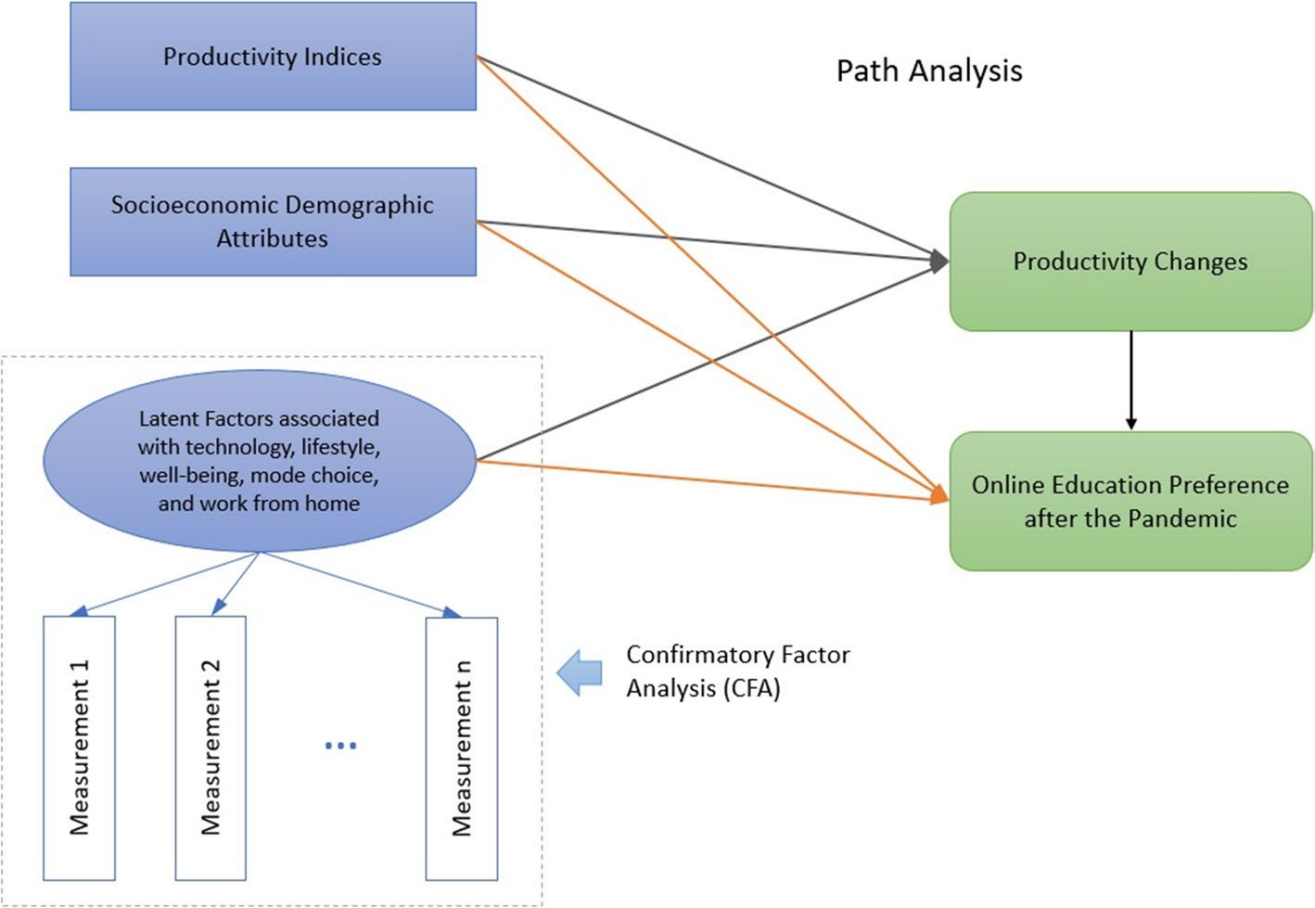
Structural equation model (SEM) path diagram

For modeling purposes, a structural equation model (SEM) seems to be the most appropriate. First, SEMs enable the simultaneous prediction of multiple endogenous variables. Second, they allow the analyst to incorporate latent factors into the model (Bollen, 1989). The literature documents successful application of structural equation models in a variety of behavioral studies (Asgari et al., 2016; Asgari & Jin, 2017, 2019; Cao, 2016; De Vos et al., 2021; Etminani-Ghasrodashti & Ardeshiri, 2015; Ingvardson & Nielsen, 2019; Lavieri et al., 2017; Mosa, 2011). SEM analysis is usually accompanied by a graphical manifestation, referred to as the “path diagram”. Path diagrams provide a better understanding of direct (and indirect) causal effects between different exogenous, latent, and endogenous variables. A path diagram demonstrating potential causal relationships is presented in Fig. 8.

The structural equation establishes the causal effects between endogenous variables (variables to be predicted) and their predictors as exogenous variables (Fan et al., 2016; Tarka, 2017). The core part of SEM analysis is to define the relationships between endogenous variables. With respect to the essence of the analysis, we tried different endogenous variables as well as different causal effects. In view of variable selection, we focused on 3 variables: pre-pandemic online class adoption (binary variable), productivity change during COVID-19 (ordered variable), and post-pandemic preference (ordered variable). Different causal effect relationships were tested, and the model was evaluated based on chi-square value and other absolute/comparative fit indices (Table 3 in the Appendix).

Our evaluation revealed that the best fit is obtained when productivity has a direct causal effect on post-pandemic preference (pre-pandemic adoption was removed from endogenous variables). In view of exogenous variables, a number of socio-economic and demographic attitudes were obtained from the questionnaire. Attitudinal preferences were extracted from Likert scale questions and were consequently converted into meaningful factor scores through CFA.

In presence of ordinal endogenous variables, the structural equation could be written as shown in Eq. 1 below.1where

ɳ^*^ = m × 1 vector of endogenous variables, here including latent functions of productivity change and preferred online class frequency after the pandemic, (m = 2)

X = q × 1 vector of observed exogenous variables, including socioeconomic and demographic attributes, and latent attitudinal factors,

 = m × 1 vector of error terms,

*β* = m × m coefficient matrix of direct effects among endogenous variables,

Φ **=** m × q coefficient matrix of direct regression effects of X on *ɳ*^∗^,

A maximum likelihood approach is the most popular method to estimate SEM parameters when endogenous variables are compatible with certain prerequisite assumptions, i.e. they follow a continuous multivariate normal distribution (Bollen, 1989; Mîndrilă, 2010; Muthén & Muthén, n.d.; Rhemtulla et al., 2012). When assumptions are not met, (e.g., in our case endogenous variables are ordered in nature), a slightly modified approach known as the Diagonally Weighted Least Squares (DWLS) is introduces and suggested in the literature (Asgari & Jin, 2017; Mîndrilă, 2010; Muthén & Muthén, n.d.; Rhemtulla et al., 2012).

The DWLS approach incorporates additional steps to convert ordered endogenous variables *η* into latent continuous variables *η*^∗^ and estimates thresholds as well as polychoric correlations (Bollen, 1989). The SEM model Parameters are then estimated by minimizing the weighted least squares fit function *F*_*WLS*_ (Muthén, 1984):

2 $${F}_{WLS}={\left[S-\sigma \left(\theta \right)\right]}^{\prime }{W}^{-1}\left[S-\sigma \left(\theta \right)\right]$$

where

*θ*= SEM Model parameters

*S*= Vector of sample statistics (i.e., threshold and polychoric correlation estimates)

*σ*= Vector containing model-implied elements of Σ(θ)

*W*= Weight Matrix

## Model results

As explained in the methodology section, our SEM model tries to predict productivity change during the pandemic situation as well as to forecast people’s preferences towards taking online classes once the outbreak is over. Such analysis allows us to identify and assess which parameters and in what way affect people’s online education preferences due to unexpected closure of in person classes during the COVID-19 government lockdown situation.

The SEM model could be decomposed into two different sub-models: The measurement model constructs latent factors based on the set of attitudinal questions in the questionnaire while the structural model establishes the causal relationships among exogenous variables, attitudinal factors and outcome endogenous variables.

Factor analysis was applied to a set of attitudinal questions in view of lifestyle, well-being, and telecommunications preferences. Responses were initially measured on a 5-scale range (strongly disagree, disagree, neutral, agree, strongly agree) and then converted into orthogonal factors using confirmatory factor analysis (CFA) with principal method. Table 1 presents the results of the measurement model. Two distinct latent factors are identified that showed significant impacts on the endogenous variables (productivity change during the pandemic and preferences for online classes after the pandemic). The First construct implies people’s attitudes toward online education, named as “pro-online learning”. The second factor indicates unsuitability of alternative locations (compared to a conventional office or school) towards work or study, named as “anti-remote location”.

**Table 1:** Results of the measurement model

	Factor 1 (Pro-online learning)		Factor 2 (Anti-remote location)	
Attitudinal Question	Coeff.	Z	Coeff.	Z
Online learning is a good alternative to high school- and college-level classroom instruction	1			
Online learning is a good alternative to elementary-level classroom instruction	1.039	12.22		
Online learning is a good alternative to extra-curricular activity instruction	0.917	10.2		
Working at home may increase family conflicts			1	
It is hard to get motivated to work away from the main office			0.949	6.44

It should be noted that we also tested a variety of other attitudinal variables in the SEM, such as attitudes toward technology, teamwork, and social interactions, but none of them exhibited significant impact in the model.

Table 2 presents the results of the structural equation model, with productivity change and online education preference after the pandemic being the endogenous variables to be predicted. In view of productivity change, our model is highly affected by the positive or negative factors affecting productivity (as discussed earlier in Figs. 5 and 6). Table 2 shows that ‘difficulty to communicate with other students’, ‘difficulty to communicate with professors’, and ‘lack of required equipment and technology’ had negative impacts on study productivity during the pandemic. On the other hand, the fact that students had more control of their time and could manage it more efficiently had a positive impact on their productivity.

**Table 2:** Results of the structural equations

		Endogenous variables			
		Student Productivity Change		Online Classes	
				After covid-19	
		Coeff.	Z	Coeff	Z
Generation	Generation X			0.543	2.388
Annual Income	$125 k–150 k	−0.955	−2.658		
Gender	Female	−0.321	−1.948		
Productivity factors	Productivity decrease: Difficult to communicate with other students	−0.369	−2.041		
	Productivity decrease: Difficult to communicate with professor	−0.663	−3.865		
	Productivity decrease: Equipment and technology not available at home	−0.541	−2.177		
	Productivity increase: More efficient time management at home	0.501	2.863		
Production change	Significant increase			0.271	4.7
Latent attitudinal factors	Home environment not suitable for work/study	−0.276	−3.596		
	Supportive of online education	0.239	3.737	0.181	3.184
Thresholds	stu_prd_chg|t1 (significant decrease- decrease)	−1.046	−6.417		
	stu_prd_chg|t2 (decrease – neutral)	−0.21	−1.362		
	stu_prd_chg|t3 (neutral- increase)	0.461	2.934		
	stu_prd_chg|t4 (increase- significantly increase)	1.604	7.199		
	ecl_prfr_nw|t1 (normal- less than normal)			−1.044	−6.297
	ecl_prfr_nw|t2 (more than normal – normal)			−0.569	−3.619
	ecl_prfr_nw|t3 (equal to pandemic – less than pandemic)			0.168	1.073
	ecl_prfr_nw|t4 (more than pandemic-equal to pandemic)			0.672	4.035
Goodness of fit measures	chi-sq = 64.83, df = 53, cfi = 0.962, rmsea = 0.032				

In terms of socio-economic and demographic attributes, income and gender were the only significant variables affecting productivity change. In this regard, a negative impact of high income (between $125 k-$150 k) was observed on students’ productivity. This might stem from the fact that people with higher incomes are full-time employees with hectic professional schedules, which could potentially interfere with their school rhythms and productivity. Also, females seemed to be more likely to experience lower productivity during the pandemic compared to males. This might be related to a higher load of family responsibilities on women’s shoulders at home such as cooking, taking care of kids, etc.

Income and gender have been historically explored in the literature in the context of online education. In view of the former, higher income is usually perceived as higher level of access to the required infrastructure and therefore, is positively associated with online education outcomes (Brown et al., 2016; Goudeau et al., 2021; Howard & Massanari, 2007; Jaggars, 2011). Mixed results are documented when it comes to gender. Some studies reported insignificant effects of gender (Lu et al., 2003, Astleitner & Steinberg, 2005, Yukselturk & Bulut, 2007, 2009, Sierra & Wang, 2002, Al-Azawei et al., 2017) while others documented that female students had a better perception of distant learning, more aligned to their academic goals and values, and usually outperformed their male counterparts (Ashong & Commander, 2012; Chyung, 2007; Dabaj, 2009; Price, 2006; Rovai & Baker, 2005). Interestingly our findings show contrary directions when compared to the literature. This might be due to couple of underlying reasons: First, it might indicate that the emergency situation created by the pandemic is not comparable to the normal pre-pandemic conditions. In particular, and based on gender, females are more likely to be overwhelmed by school shutdowns and the increased responsibility of childcare or other relevant housekeeping duties. They might also be more emotionally distressed about the uncertain situation, which lowers their productivity compared to their male counterparts. Also, it might signify that attitudes are outperforming demographic impacts. For instance, while income is mainly associated with infrastructure access, this aspect has already been accounted for in the model through several direct attitudinal and perceptional questions. Hence, it is reasonable to assume that some of the socio-economic and demographic attributes might be affected in terms of their direction of impact or their significance in the model.

In view of latent factors, “pro-online education” showed positive impacts on productivity change, while “anti-alternative location” showed negative impacts. This indicates that those who were more supportive of online education, were more likely to experience increased productivity during the pandemic, while those who did not think home as suitable environment for work or study were more likely to experience decreased productivity during the pandemic.

In terms of the preferences of taking online classes after COVID-19 is no longer a threat, those who experienced positive changes in productivity during the pandemic were more likely to prefer increased frequency of online classes, as expected. The same stands for those with *pro-online learning* attitude. Again, none of the socio-economic and demographic factors (except for Gen-Xers) showed significant contribution to their preferences. The positive impact of Gen-Xers is likely to stem from the fact that they are usually full-time workers who benefit from online programs to accommodate both work and school activities. Hence, they prefer to continue with online programs at least as frequent as before. This inference also confirms the findings from a recent study, where Generation X showed the highest satisfaction for course design, course delivery, preference for mode of delivery and total overall satisfaction with online learning (Yawson & Yamoah, 2020).

It should be noted that since there is a positive causal effect from productivity change to online adoption preferences, all the exogenous variables affecting productivity change also had indirect impacts on their future preferences in the same direction. For instance, being a female, suffering from lack of sufficient interactions with the instructor or other students, or those who did not find home an encouraging environment for work or school also discouraged their preferences for taking online classes in the future but in an indirect manner (through reductions in productivity). The only feature with both direct and indirect impacts on online adoption is the *pro- online education* attitude, which adds up to a total effect of (0.181 + 0.271 * 0.239 = 0.245). Which means a pro-online education attitude will increase the odds of taking online classes by 27.7% (*e*^0.245^ − 1) compared to those without this attitude.

## Policy implications

This study shed light on students’ perceptions and experience of emergency remote learning during COVID-19. The results from this study can also help provide insights on how to make online education more efficient and more productive based on what we have learnt through the pandemic.

As our analysis shows, most socio-economic and demographic variables have generally no significant impacts on online education experience or future preference. This is somewhat reasonable, indicating that people have now become more aware of the benefits and challenges involved in distant learning, and that the attitudes and perceptions play a more important role in their decisions rather than socio-economic or demographic attributes. We might expect to see a positive impact of higher income households on online education adoption or higher education productivity (due to monetary costs of required equipment) but in practice our model shows a negative impact for high income groups, which might be attributed to their work status and the challenge in balancing work and school activities.

In view of perceptions toward online education, one can infer that better time management is one of the top benefits of online classes, however usually there are certain number of conflicts involved. In this regard, lack of sufficient communication between students and with the instructor is a major problem, which is expected to be solved or at least improved when students are given sufficient education about the online system and how everything works in a virtual environment. Instructors can provide tutorials regarding the specific virtual environment being used for their classes, or try to enhance virtual communications through emails, social media, and establish regular meetings to support students. In view of equipment and technology, internet companies should continue providing deals and discounts for students. Tech companies are recommended to provide simpler and more user-friendly virtual conference/meeting applications/software as well as to provide simple-language tutorials for their audience.

Our analysis revealed that among different attitudes, pro-online education and viewing home or alternative locations as suitable environment for work/study are the most critical attitudes. Being an online education fan directly encourages both productivity and future preference. Considering that this attitude highly relies on familiarity and usage of online educational applications, authors recommend that schools and institutes should invest in tutorials or training programs for students to get familiar with online educational programs and encourage students to participate. This will help increase the familiarity of both instructors and students in a practical environment and will help identify and resolve many of the challenges involved in a timely manner. In terms of alternative locations for remote study, there are many different factors that could contribute to home environment not being suitable for work/study, ranging from physical factors (such as presence of kids or seniors at home, presence of other workers or students, lack of work/study space, etc.) to emotional/mental reasons (feeling of isolation, lack of motivation, difficulties to concentrate, etc.). Suggestions and guidelines may be helpful for students to set up their study area and routines at home. Efforts may also incorporate remote work/study considerations into floor layout design for houses.

## Conclusion

This paper presented the results of a recent survey on students’ perception of benefits and challenges involved in distant learning during the pandemic. Data was collected in May 2020 in the state of Florida through an online survey. In particular, this research focused on college students.

Our initial analysis showed that only around 18% of the respondents believed that their education quality has increased compared to the normal conditions before the pandemic. Approximately 52% of the students stated that they perceived the online education quality to be lower than normal in-person conditions. A similar trend was observed in view of students’ productivity. The majority of respondents (around 60%) indicated that their productivity was lower or significantly lower than traditional approaches compare to before COVID-19. Different positive and negative factors were associated with productivity changes. In view of negative factors, many stated that they had more distractions at home, or it was difficult for them to maintain appropriate levels of communication with the instructor as well as other students. Some other mentioned that they lacked comfortable workspace at home while others complained about conflicts with home responsibilities. On the other hand, many respondents appreciated more efficient resting time, more casual work environment at home and no commuting time as the most popular positive advantages of online education. In view of future preferences, around 61% of the respondents stated that they would prefer more frequent online education compared to normal (before the pandemic) conditions.

In order to identify the contributing factors and assess their impacts on the future adoption of online education, a SEM was developed to predict productivity change and future preference towards online education. In view of productivity, our model showed that high income individuals and females would have lower productivities compared to other sociodemographic groups. Lack of communication with the instructor and students as well as lack of required equipment/technology were also significant barriers against productivity increase. On the other hand, better time management at home turned out to be positively affecting online education productivity (and future adoption). Among different attitudes, pro-online education was likely to increase productivity, while those who did not find home as a suitable environment for work/study (due to distractions or other conflicts) suffered lower productivity during the pandemic. As expected, a direct positive impact is observed from productivity increase to future preferences of taking online classes, indicating an indirect impact (with a similar sign) associated with all the aforementioned factors on future preferences. The only demographic variable with a direct impact on future preference is Gen-Xers, who were likely to adopt more of online education in the future, probably because of their activities in professional and family responsibilities and their effort balance school, work and family.

This study and similar studies are expected to shed light on the status of online education as the new norm during the pandemic and also provide further insights on whether (and how) the pandemic experience could be used to understand students’ needs and accordingly improve the quality of online education programs to suit their needs.

Finally, it should be noticed that this study is subject to a number of limitations. In particular, the survey was conducted at the early stages of the pandemic outbreak, where the public were reasonably shocked and were not fully adapted to the new norms. As the pandemic has lasted more than a year and might have long-lasting effects, one future research avenue would be to reassess individuals’ perceptions and attitudes as time passes by, potentially through a second wave of survey. Also, while this study focused on the demand side (i.e., students), similar studies could be conducted on the supply side (i.e., schools, instructors, and principals) to further explore their needs for a successful deployment of online programs.

## Data Availability

Available upon request.

## Appendix

**Table 3:** Different combination of effects among potential endogenous variables

Structure	chisq	df	cfi	chisq/df
Current structure (causal effect from productivity to future preference)	14.87	11	0.988	1.351818
Current structure + causal effect from pre-pandemic online education frequency to productivity	42.46	17	0.923	2.497647
Current structure + causal effect from pre-pandemic online education frequency to productivity and future preference	40.317	16	0.927	2.519813
correlation between future preference and productivity	24.94	11	0.959	2.267273
correlation between pre-pandemic online education frequency, future_preference, and productivity	26.88	15	0.967	1.792

Table 3.
